# Supplementary material for: Are carbon emissions trading and green financial instruments synergistic? -Comprehensive quantitative research based on content analysis
Source: PLoS One. 2024 Mar 7;19(3):e0298601. doi: 10.1371/journal.pone.0298601 (PMC10919601; doi:10.1371/journal.pone.0298601)
Supplement: S1 Data — (ZIP) [file pone.0298601.s001.zip › Data/地级市Z_F工作报告.zip!/║╙─╧/╚2├┼╧┐╩╨/└┤╫╘╣1⁄2╓┌║┼í╛┬φ┐╦╩2╛▌═°í┐_2011.docx]

各位代表：

　　现在，我代表市人民政府，向大会作政府工作报告，请予审议，并请各位政协委员提出意见。

　　一、2010年及"十一五"工作回顾

　　刚刚过去的2010年，是实施"十一五"规划的最后一年，也是我市经济社会发展极不平凡的一年。面对复杂形势和严峻挑战，在省委、省政府和市委的正确领导下，在市人大、市政协的监督支持下，市人民政府团结和带领全市人民深入贯彻落实科学发展观，坚持"四个重在"实践要领，牢牢把握"调结构、促转型、增效益"这一主线，继续深化项目建设年和企业服务年活动，抢抓机遇，顽强拼搏，开拓创新，锐意进取，圆满完成了市五届人大五次会议和"十一五"规划确定的各项目标任务，经济社会保持了平稳较快发展的良好态势。

　　（一）国民经济高位运行。初步核算，全市生产总值完成874亿元，比上年增长15.2%；工业增加值完成562亿元，增长18.5%；全社会固定资产投资完成677.5亿元，增长22.4%；社会消费品零售总额达203亿元，增长18%；城镇居民人均可支配收入达到15032元，农民人均纯收入达到5787元，分别增长11.6%、14.7%。

　　（二）跨越发展能力不断增强。坚定不移抓好重点项目和产业集聚区建设，市"双百工程"完成投资344.6亿元，101个项目建成，126个在建项目加快推进；大唐发电三期获国家发展改革委核准，540个项目进入省"十二五"规划盘子；省督办联审联批事项全部办结，居全省第一。全市7个产业集聚区完成投资242.8亿元，新开工项目119个，投产77个，在建125个，三门峡产业集聚区、义马煤化工产业集聚区被确定为全省新型工业化产业示范基地。扎实开展大招商活动，签约项目135个，总金额达1134.3亿元，履约率、开工率分别达94.8%、67.4 %；全市实际利用外资3.98亿美元，比上年增长51.2%，绝对值居全省第三；实际到位省外资金114.4亿元，增长34.7%，增幅居全省第三。通关机构建设初见成效，河南出入境检验检疫局三门峡办事处挂牌开办，国家铝及铝制品质量监督检验中心主体完工，三门峡海关有望近期获批，外贸出口完成1.04亿美元，增长28.2%，超额完成省定目标。加强区域经济合作，积极参与中原经济区建设，区域竞争力进一步提升。

　　（三）经济发展质量和效益明显提高。工业效益整体良好，规模以上工业实现利税259亿元，比上年增长50.9%。财政保障能力持续增强，全市财政总收入81.7亿元，地方财政一般预算收入49.7亿元，增长19.8%。全市存款余额625.52亿元，较年初增加116.6亿元；贷款余额340.2亿元，较年初增加75.92亿元，增长28.73%，增幅全省第一。落实最严格的节约集约用地制度，连续12年实现耕地占补平衡有余；电力供应安全稳定，售电量达125亿度。大力发展循环经济，12家资源节约综合利用企业通过省级认定。强力推进节能减排，COD、二氧化硫减排达到目标要求。县域经济发展迅速，综合实力不断提升。

　　（四）经济结构战略性调整扎实推进。五大支柱产业持续增长，自主创新体系建设加快。速达纯电动汽车下线运行，被确定为全省电动汽车生产基地和电动汽车示范运营城市；恒生柠檬酸金钾等技术领先项目开工或建成。3家企业研发机构创建为省级院士工作站， 28家企业被认定为省高新技术企业。特色农业不断壮大，果品总产16.6亿公斤，烟农纯收入3.63亿元，烟叶收购保持全省第一；拥有规模以上农业龙头企业150家，省级重点农业龙头企业17家；地理标志保护产品9个，被批准为全国创建农业标准化综合示范市。现代服务业快速发展，大中海商业文化广场、大鹏酒店二期、海联大酒店等项目扎实推进。洛阳银行入驻我市，市商业银行设立3家分行。三省八市旅游联盟开展深度合作，黄河旅游节暨投洽会对外影响进一步扩大，接待入境游客5.3万余人，实现旅游总收入94.28亿元。

　　（五）统筹城乡发展力度加大。"三纵四横"交通枢纽建设加快推进，三淅高速、连霍高速扩建等重点项目开工。改造建设陕州大道、上官路等8条城市道路，集中供热二期等民生工程顺利完成，城中村和旧城改造稳步推进；创建国家森林城市、卫生城市扎实开展，城市功能逐步完善。建成农村公路380公里，完成造林绿化48.7万亩，新增和改善灌溉农田3.19万亩，治理水土流失面积84平方公里，解决农村10.19万人饮水安全；完成搬迁扶贫880户、3762人，转移农村剩余劳动力36万人，实现劳务收入29亿元。新农村建设成效显著，40个新型农村住宅社区建设试点累计完成投资13亿元，建成10034套，入住4100户，18个农村社区基础设施配套到位，城乡一体化进程明显加快。

　　（六）社会民生进一步改善。扎实推进省十项民生工程和市十件实事，市文体中心体育场封顶，市中心医院病房楼投入使用。保障性住房建设进度加快，建成廉租房4331套、21.66万平方米；建设经济适用房777套，竣工3.91万平方米。社会保障体系不断完善，新增城镇就业人员5.89万人、城镇下岗失业人员再就业2.73万人，城镇登记失业率3.24%；新农合参合率达97.89%。加快发展文化教育事业，市外国语高中正式招生，三门峡职业技术学院在校生达2.1万人，仰韶文化博物馆主体竣工。食品药品安全监管加强，社会救助力度加大。人口计生和人民防空工作被评为全国先进。扎实开展安全生产，应急保障能力增强，成功处置中石油陕西柴油泄漏事件。抗洪救灾取得重大胜利，灾区倒房重建如期完成，荣获全国防汛抗旱先进集体。

　　（七）民主法制和精神文明建设得到加强。自觉接受市人大及其常委会的监督，支持市政协履行职能，认真办理人大代表建议59件、政协委员提案224件，满意或基本满意率达100%。深化行政管理体制改革，市、县政府机构改革全面完成。坚持依法行政，加强政府自身建设，反腐倡廉工作取得新成效。强化精神文明建设，公民道德水平和文明程度全面提升。坚持"三具两基一抓手"，深入推进"两转两提"，认真开展"创先争优"活动，干部群众干事创业、团结进取的氛围更加浓厚。加强群众工作和平安建设，成为全国社会管理创新综合试点市。大力开展国防教育，扎实做好双拥工作，驻市解放军、武警和预备役部队在支持地方经济建设、处置突发事件等方面作出了重要贡献。民族、宗教、对台、外侨、统计、审计、档案、史志、体育、气象、地震、无线电、黄河河务、妇女儿童、残疾人、老龄等工作取得新成绩。四川江油灾后援建任务圆满完成，及时启动对口援疆工作。

　　各位代表！随着 2010年各项任务的圆满完成，标志着"十一五"规划确定的目标胜利实现。这一时期，国际金融危机严重冲击，资源环境约束加紧，结构性矛盾日益突出，经济形势复杂多变。面对压力和挑战，我们见事早、行动快、措施实，积极实施"一体两翼"经济刺激战略，果断采取"四区"建设、抓好六大载体等有效举措，克难攻坚，化危为机，在逆境中保持经济平稳较快增长，经济社会进入快速发展的崭新阶段。

　　--这五年是经济社会持续发展、综合实力跃上新台阶的五年。国民经济连续五年实现较高增速，生产总值年均增长15.3%，增速比"十五"时期提高4.3个百分点，人均生产总值超过39200元（折5790美元）。地方财政一般预算收入五年完成178.9亿元，年均增长24.5%，高出目标8.5个百分点。

　　--这五年是实施经济结构战略性调整、加快发展方式转变的五年。扎实推进经济结构调整，产业结构不断优化，五大支柱产业增加值占工业增加值的比重达80%，二、三产业比重达92%，比"十五"末提高2个百分点；自主创新能力不断增强，科技进步对经济发展的贡献率达52.7%。万元生产总值能耗累计下降21%。

　　--这五年是基础设施逐步完善、城乡面貌显著变化的五年。抢抓扩内需机遇，加快重大基础设施建设，全社会固定资产投资完成2154亿元，比"十五"增加3.5倍。交通基础设施建设投资38.6亿元，公路通车总里程达9258公里；水利项目总投资20.8亿元，是"十五"的10倍。

　　--这五年是改革深入推进、开放水平大幅提升的五年。积极应对国际金融危机冲击，坚持深化改革，全面扩大开放，通关机构建设步伐加快。实际利用外资12.2亿美元，比"十五"增加10.1亿美元；实现外贸出口6.44亿美元，比"十五"增加2.05亿美元，优势产品远销欧美等海外市场。

　　--这五年是社会事业全面进步、人民群众得到更多实惠的五年。民生投入明显加大，向农民发放各类补贴 17.5亿元，"十件实事"累计投资35亿元。五年新增城镇就业28.4万人，城镇登记失业率连年保持较低水平。社会事业长足发展，公共服务体系不断完善。城镇居民人均可支配收入、农民人均纯收入年均增长13.2%、14.5%，均高于"十五"平均水平。

　　各位代表！这些成绩的取得，是省委、省政府和市委正确领导的结果，是市人大、市政协大力支持的结果，是全市广大干部群众迎难而上、团结奋斗的结果。在此，我代表市人民政府，向工作在全市各条战线上的工人、农民、知识分子、干部、人民解放军指战员、武警官兵、公安民警以及社会各界人士致以崇高的敬意和衷心的感谢！

　　各位代表！这五年取得的成绩来之不易，积累的经验弥足珍贵，有四点需要继续坚持和发扬：一是必须科学发展调结构。牢牢把握发展第一要务，坚持用科学发展观武装思想，抢抓加快转变经济发展方式的难得机遇，在发展中调结构，在转型中谋发展，促进经济社会又好又快发展。二是必须持续推进保增长。紧密结合资源型城市转型升级的实际，始终坚持以经济建设为中心不动摇，坚定不移地抓好重点项目和产业集聚区建设，立足当前保增长、促发展，着眼长远打基础、增后劲，努力变资源优势为经济优势，加快建设经济强市。三是必须改革创新增活力。坚持把改革开放作为加快发展的动力源泉，依靠体制机制创新破解发展中的难题，积极招商引资承接先进产业转移，通过战略合作实现优势互补、借力发展，大力扶持民营科技企业，培育壮大高新技术产业，为经济社会跨越式发展积蓄力量。四是必须改善民生促和谐。把保障和改善民生作为发展的根本出发点和落脚点，把调结构、促转型和保增长、惠民生有机结合，不断加大民生投入，坚持办好十大实事，有效促进社会和谐稳定，为改革、发展提供坚强保障。

　　各位代表！在充分肯定成绩的同时，我们也清醒地看到，政府工作与人民群众的期待还有一定差距，当前经济社会发展还存在不少矛盾和问题：国民经济连续多年高位增长，继续保持快速增长难度加大；经济结构深层次矛盾仍然存在，经济发展面临"调结构"与"保增长"的双重压力，节能降耗形势尤为严峻；城市规模不大，"三化"协调发展动力不足，城镇化建设任务繁重；城乡居民持续增收难度加大，改善民生任务还很艰巨；政府自身建设、推动科学发展的能力需要进一步增强。对此，我们一定高度重视，认真加以解决。

　　二、"十二五"发展的目标任务

　　"十二五"是我市全面建设小康社会的关键时期，是加快跨越式发展、转变经济发展方式的攻坚时期。面对新形势、新任务，我们必须抢抓机遇、乘势而上，聚精会神搞建设，一心一意谋发展，为打造中原经济区重要支撑、区域合作示范城市和豫晋陕黄河金三角区域中心城市奠定坚实基础。

　　根据市委五届八次全会精神和市"十二五"规划纲要，今后五年我市经济社会发展的主要奋斗目标是：经济发展实现新跨越。全市生产总值年均增长12%；财政总收入年均增长12%，一般预算收入年均增长13%，力争"十二五"末比2010年翻一番；全社会固定资产投资年均增长15%以上。转型升级要有新突破。到2015年，全市特色农业产值占农业总产值比重超85%；高新技术产业增加值占规模以上工业增加值比重超过10%。民生改善再上新台阶。城乡居民收入年均增长10%；到"十二五"末，城镇化率达到55%。生态建设取得新成效。到2015年，全市森林覆盖率达53.5%，完成省定万元生产总值能耗、二氧化碳排放量、主要污染物排放量等指标，年城市环境空气质量优良天数超320天。

　　实现上述目标，必须突出三大战略重点：一是加快经济发展方式转变，保持经济平稳较快发展。必须加快新型工业化进程，强力实施"1333"工业转型升级工程，以产业集聚区为载体，大力促进传统产业高端化、新兴产业规模化，尽快形成产业结构合理、产业布局科学、产业发展集聚的现代工业体系；必须完善新型城镇化体系，大力发展现代农业，突出发展现代服务业，推动城乡统筹协调发展，促进经济结构战略性调整；必须坚持生态文明建设，加快推进自主创新，增强区域竞争力，努力建设资源节约型和环境友好型城市。二是着力改善社会民生，加快富民强市步伐。必须坚持富民优先、强市为要，着力扩大就业，加快全民创业，完善社会保障体系，稳定物价总体水平，千方百计增加城乡居民收入。优先发展教育事业，推进基本公共服务均等化，创新社会管理，构建和谐社会，在发展社会事业和改善民生方面取得更大进展。三是坚持改革开放，提升体制机制创新水平。必须把改革创新作为加快发展的动力，最大限度地解放和发展生产力。深化经济体制改革，全面推进行政管理、要素市场等领域的机制改革，努力在重点领域和关键环节取得突破性进展。坚持对内对外开放并举，扩大总量与提高质量并重，加快推进豫晋陕黄河金三角试验区建设，支撑中原经济区发展，形成全方位、多层次、宽领域的对内对外开放新格局。

　　各位代表！《三门峡市国民经济和社会发展第十二个五年规划纲要（草案）》及说明已印发各位代表，请予审议。我们坚信，有省委、省政府和市委的坚强领导，有全市人民的齐心协力、奋勇拼搏，我市"十二五"发展蓝图一定能够如期实现，为全市经济社会发展再谱新篇、再铸辉煌！

　　三、2011年工作安排

　　今年政府工作的总体要求是：认真贯彻党的十七届五中全会和省、市委经济工作会议精神，全面落实科学发展观，以加快转变经济发展方式为主线，以富民强市为中心任务，坚持调结构、促转型、保增长、惠民生，加快"一个载体、三个体系"建设，继续深化项目建设年、企业服务年和城建提速年活动，扎实推进"三化"协调发展，努力促进经济又好又快发展，为建设充满活力、富有魅力、极具竞争力的现代化城市奠定坚实基础。

　　主要预期目标是：生产总值增长12%，地方财政一般预算收入增长13%，全社会固定资产投资增长21%以上，工业增加值增长13%，社会消费品零售总额增长16%，外贸出口增长21%，城镇化率达49%，城镇居民人均可支配收入、农民人均纯收入分别增长9%，完成省定万元生产总值能耗等指标。重点抓好八个方面工作：

　　（一）突出抓好产业集聚区和重点项目建设，进一步增强跨越式发展动力。强力推进产业集聚区建设。全年投资超230亿元，其中工业项目投资超200亿元，力争全市7个产业集聚区投资增幅、主营业务收入增幅超过全省平均水平。加强基础设施建设，建设标准化厂房60.6万平方米，提升产业承载能力；加快项目建设，重点推进明彩集团年产100万色令印刷制品、义煤集团年产30万吨铝板带箔、盛源化工年产30万吨醋酸等58个投资超亿元项目，引导城区企业向同类园区集中，推动支柱产业优化升级；完善管理体制，加快投融资、土地储备、中小企业担保三大平台建设，提升发展保障能力；加快产城互动发展，提升产业规模和发展速度，创造就业岗位，促进人口转移。抓好三门峡光伏产业园、湖滨机电制造业园区等专业园区建设，提高专业化、规模化水平。坚定不移地抓好重点项目建设。继续实施"双百工程"，强力推进200个以上重大产业升级和重大基础产业、基础设施项目，总投资1000亿元以上，今年完成投资300亿元，保持固定资产投资的连续性和稳定性。全力抓好速达年产20万辆纯电动汽车及50万套核心零部件、大唐2×100万千瓦超超临界机组、义煤综能1000万方煤制气、地久矿业7000吨多晶硅一期等重点转型升级项目，高起点谋划一批科技含量高、发展前景好、事关经济社会发展全局的优质项目，加快三淅高速、郑卢高速、连霍高速扩建等重大基础项目建设，增强可持续发展动力。建立健全长效推进机制。继续落实市领导分包重大项目等制度，实行"一个领导牵头、一个法人承担、一个部门负责、一个小组落实"工作机制，健全督导推进体系，完善考核奖惩体系，营造激励竞争的浓厚氛围。

　　（二）必须坚持在发展中调整结构，切实加快转变经济发展方式。巩固提升优势产业。做大做强五大支柱产业，重点改造提升煤化工、黄金生产及加工、铝及铝精深加工等三大传统优势产业，力争2012年主营业务收入达1200亿元，两年增加450亿元。按照基地化、规模化要求，大力发展精深加工，加快煤化工产业链条向高端延伸；推进黄金精炼、工艺品生产及新产品研发，使矿产资源向优势企业集中，提高黄金生产及加工产业的规模化程度；优化铝工业产业布局和资源配置，在氧化铝资源就地转化和铝精深加工方面实现突破。大力推进战略新兴产业。积极培育新能源汽车、生物、新材料、新能源四大先导产业，加快速达纯电动汽车项目建设，抓紧完成200辆示范运行样车装配下线并投放市场；建成清源风电场二期、鞍子山风电场，努力成为全省最大风电基地；抓好恒生柠檬酸金钾扩建、兴邦纳米离子膜以及光伏产业等项目进度，推动战略先导产业向高端化、规模化发展，2012年主营业务收入达200亿元，增加150亿元。强力发展汽车及零部件、装备制造、食品三大高成长性产业，推进高档轿车轮毂、铝合金整体式车厢等汽车零部件产业发展，在水工机械、大型压力容器、检测量仪、数控机床等领域加快开发成套装备和产品，用两年时间培育形成一批百亿产业集群，主营业务收入超320亿元。加强苹果加工、畜牧养殖基地建设，发展一批特色优势食品产业集群，争取2012年主营业务收入超100亿元。加快发展现代农业。继续稳定粮食生产，大力发展特色农业，全年果品总产17亿公斤，食用菌栽培1.2亿袋以上，中药材种植面积23万亩，种植烟叶25万亩，实现苹果、浓缩果汁出口新突破。加快农业产业化发展，力争规模以上农业龙头企业达160家，农业专业合作社发展到320家以上。培育壮大现代服务业。抓好大中海商业文化广场、湖滨汽车城等重大商贸项目和湖滨区核心商圈建设，加快年交易10万吨大型果蔬冷链物流集散中心等项目建设，推进食品冷链、铝制品等特色物流园区和综合园区发展。继续推进银行业改革，支持市商行改制，鼓励发展域外金融业务，加快农商行组建步伐，积极引进1家股份制商业银行。加快信息化进程，建设"无线城市"。抓好沿黄生态旅游经济带等8大旅游项目规划编制和对外招商，全面推进函谷关等20个景区和基础设施建设，年内旅游招商突破30亿元，新增4A级旅游景区1-2家、4星级以上旅游饭店1-2家，实现旅游综合收入116亿元，增长30%。高水平办好第十七届黄河旅游节暨投洽会，打造国内外知名的黄河文化旅游品牌。

　　（三）强力推进城乡统筹，全面提升"三化"协调发展水平。壮大中心城市规模。大力实施中心城市带动战略，高标准规划建设城市新区，重点抓好义乌商贸城、迎宾大道沿线开发，加快三门峡南站起步区和三灵快速通道建设；做好老城区优化提升，强力实施"双十"工程，集中抓好十大市政基建项目、十大城中村和旧城改造项目，促使209国道市区段沿线旧城改造取得实质性进展，有效推动新老城区功能互补、发展互动，促进湖滨区、陕县、灵宝市组团发展。建设生态宜居城市。加强城市精细化管理，加大重点部位环境整治，提升城市管理水平，创建河南省最佳宜居城市。加快国家森林城市创建步伐，率先建成全省林业生态建设先进市。深入开展国家卫生城市创建活动，加强公共卫生体系建设，提高群众健康水平。完善城乡基础设施。积极推进运三铁路、连霍高速扩建等重大交通项目建设，加快形成"三纵四横"交通枢纽中心。强力实施城乡建设三年大提速行动计划，投资200亿元加强城市基础设施建设，改造崤山东路、大岭南路等城市道路，配备电动汽车充电站（桩），满足电动汽车发展需要。加大公共服务设施建设力度，开工建设市文化公园，确保市文体中心体育场在第十七届黄河旅游节暨投洽会开幕式投入使用。优先发展城市公共交通，推进农村公路畅通工程，实现城乡客运交通一体化建设。加大财政投入力度，加强农田水利基本建设，做好新一轮病险水库除险加固，加快山口水库建设进度，完成水土流失治理面积80平方公里，新增有效灌溉面积3万亩、旱涝保收田3万亩、节水灌溉面积3万亩，解决农村8万人安全饮水问题。集中抓好全市防汛指挥系统及山洪灾害预警体系建设，提高防灾避灾水平。积极推进城乡一体化。义马、渑池城区对接要有新进展，推进灵宝北区和西区开发，抓好卢氏生态建设。重点发展310国道沿线和市域周边乡镇，扶持区位优势明显、发展潜力较大的10个中心镇、50个村作为城镇化示范带动村镇。抓好40个省市新型农村住宅社区和35个县级示范社区建设，促进城乡协调发展。

　　（四）认真抓好经济运行调控，努力保持经济平稳较快增长。不断深化企业服务。加大市属重点企业服务力度，坚持领导分包、巡回服务等制度，引导关联企业加强协作，推动企业服务工作常态化。积极推进企业战略重组，培育产业链条完整、竞争优势明显的大型骨干企业或集团，提高企业抗风险能力。大力支持非公有制经济发展，积极服务中小企业和微型企业快速成长。切实加强经济要素保障。建立经济运行协调机制，加强对经济运行的监测、研判、预警，强化产销、产需、供需、银企对接等平台建设，提高煤电油运等生产要素保障能力。全面加快电网建设，进一步提高安全供电和优质服务水平。严格落实耕地保护制度，扎实开展节约集约用地模范县（市）创建工作，依法加强重点建设用地保障，全力开展矿产资源整合和矿山地质环境治理恢复。扎实做好财政金融工作。完善税收征管机制，堵塞税收征管漏洞，确保应收尽收。强化财政支出监管，用足用好财税政策，优先保证民生投入。完善信用担保体系建设，科学增加有效信贷投放，确保贷款增速高于全省平均水平。努力保持物价总体稳定。落实价格监管措施，严厉打击囤积居奇、哄抬价格等不法行为，有效防止通胀预期上升，保持主要农产品和重要生产资料价格基本稳定。建立完善最低生活保障、失业保险标准与物价上涨挂钩的联动机制，保障低收入群体基本生活需要。全面落实节能减排措施。抓好公共机构和电力、有色金属冶炼等重点行业的节能减排，淘汰过剩和落后产能，深入挖掘节能潜力。大力发展循环经济，鼓励企业技术改造，支持企业开展废渣、废水、废气的综合利用。加强环境保护，做好重点区域和行业环境综合整治，全面完成各项节能减排任务。

　　（五）扎实推进改革创新，不断激发经济发展活力。壮大城区经济规模。集中精力抓好市级经济发展，按照利益共享、责任共担原则，统筹包括湖滨区、开发区、产业集聚区、陕县县城在内的中心城区发展，健全税收分成等利益分配机制，科学规划产业和项目布局，积极发展"飞地经济"，促进城区经济跨越式发展。增强县域经济实力。强化指导服务职能，加大支持力度，能放的权力坚决下放，能给的政策坚决落实，增强县域经济的发展活力。发挥各县（市、区）主体作用，突出县域特色，构筑优势产业体系，积极在全省晋位争先，快速提高县域经济发展水平。推进自主创新建设。实施科技兴市和人才强市战略，加强科技创新体系建设，加快民营科技企业发展，培育1-2家省级院士工作站、1-2家省级重点实验室、2-4家省级工程技术研究中心和3-5家拥有自主知识产权的创新型企业；加大科技创新扶持力度，实施义马年产3000万套锂离子电芯等高新技术产业项目，培育高新技术企业5-8家，力争高新技术产业增加值增长15%，科技进步对经济增长的贡献率达54%。深化农村体制改革。继续推进集体林权制度、县级供销社体制改革，建立健全农技推广、动植物疫病防控、农产品质量监管等公共服务机构，加快推进农村土地流转，完成土地流转面积35万亩。

　　（六）全方位扩大对外开放，有效增强区域经济竞争力。提高招商引资实效。坚持"走出去、请进来"，加大招商引资力度，以开放促招商，以招商促发展。抢抓承接国内外产业转移和加快资源配置的战略机遇，以长三角、珠三角、闽台地区为重点，积极开展对接洽谈，实行链式招商、专业招商、以商引商，招大商、招好商、招高商，逐步实现招商引资工作向市场运作转变。完善项目协调、跟踪督查、评价奖惩等工作机制，发挥协调督查联席会议作用，抓好签约项目跟踪落实，全年实际利用外资4.86亿美元，到位省外资金137.3亿元。加快通关机构建设。建成国家铝及铝制品质量监督检验中心，开工建设三门峡海关，申报、筹建省级危险化学品包装容器检测中心、铬盐化工检测站等项目。实施名牌战略、标准化战略和质量安全战略，培育出口优势企业，引进出口潜力企业，抓好轮毂、果汁等优势产品出口，支持高科技产品出口，全年实现外贸出口1.25亿美元。加大战略合作力度。围绕铝精深加工、新能源开发等优势领域，瞄准世界、国内500强和行业50强企业，有针对性开展经济合作，不断提升合作层次和水平。巩固政银企战略合作成果，确保工农中建四大银行签约资金落实到位。加强国际友好往来，务必在经贸合作上取得实质性成果。强化区域经济合作。积极参与中原经济区建设，加强与洛阳、济源经济合作，共同构建支撑中原经济区豫西板块；加强豫晋陕黄河金三角区域经济合作，增强区域竞争力。

　　（七）切实保障和改善民生，全面发展社会事业。突出抓好省十项民生工程和市十大实事，切实维护好群众利益。促进城乡居民增收。实施积极就业政策，促进以创业带动就业，发放小额担保贷款1.35亿元以上，确保全年城镇新增就业4万人，下岗失业人员再就业2万人以上，动态消除"零就业"家庭。落实粮食最低收购价政策，大力发展生态观光农业和"菜园子"、"菜篮子"经济，确保肉类总产达8.1万吨、蔬菜8.2亿公斤、水产品9000吨。积极发展劳务经济，农村劳动力技能培训2.1万人，阳光工程培训8000人，打造劳务品牌，促进农民增收。强化社会保障体系建设。完善最低生活保障制度，全面实现城镇基本医疗保险即时结算，稳妥推进医疗保险市级统筹。全市企业养老、失业、城镇职工医疗、城镇居民医疗、工伤保险参保人数分别达到26万人、22.5万人、30.4万人、31.3万人、15万人。积极改善群众住房条件，建设公共租赁住房2000套以上、廉租住房1850套，建立满足不同收入群体需要的城镇住房保障体系。大力发展社会事业。加大教育投入，整合教育资源，推动义务教育均衡发展；加快职教攻坚进度，努力提升高等院校办学层次。深入开展群众性精神文明创建活动，大力发展文化体育事业，加快虢国、仰韶、老子三大文化产业园区建设，支持渑池县办好纪念仰韶文化发现90周年系列活动。继续抓好人口计生工作，人口自然增长率控制在6.48‰以内。加强食品药品安全监管，加快市中心血站和医疗机构标准化建设，构建农村医疗卫生服务网络，缓解群众住院难、看病难问题。加大社会化拥军力度，连创全国双拥模范城。继续做好民族、宗教、统计、史志、黄河河务、审计、事管、盐业、地震、人防等工作。扎实做好支援新疆农十三师柳树泉农场工作。

　　（八）创新社会管理，维护社会稳定。坚决抓好安全生产。牢固树立安全发展理念，全面开展隐患排查治理，强力推进煤矿兼并重组，加强非煤矿山、道路交通、消防等重点领域、重点行业、重点区域监管，杜绝一切违法违规生产行为，为经济发展提供安全环境。全力维护社会稳定。全面排查矛盾纠纷，强化信访源头治理。坚持信访周会审、领导包案等制度，深入开展"群众工作日"活动，推行领导接访工作新模式，深化信访代理、流动调解机制，畅通群众诉求渠道，提高案件办结率和满意率。深入开展平安建设。加强社会治安综合治理和科技防控体系建设，积极开展全国社会管理创新综合试点工作，严厉打击各类刑事犯罪，增强人民群众安全感，争创全国综治"长安杯"。加快应急体系建设，妥善处理突发公共事件，保持社会和谐稳定。

　　四、加强政府自身建设

　　做好今年各项工作，必须以领导方式转变加快发展方式转变，坚持"三具两基一抓手"，深入推进"两转两提"，扎实开展"创先争优"活动，建设人民满意政府。

　　（一）必须解放思想。坚持更新观念、与时俱进，进一步解放思想，自觉把思想从落后的观念、传统的做法和体制的束缚中解放出来，使思想和行动更加符合科学发展观的要求，以奋发有为的精神状态，不断开创各项工作新局面。强化机遇意识、责任意识、忧患意识，始终保持强烈的事业心和责任感，突出运作，狠抓落实，坚持说到做好，确保工作落到实处、取信于民。

　　（二）切实转变职能。加强和改善政府对经济社会事务管理，努力实现政府职能向创造良好环境、提供优质公共服务和维护社会公平正义转变。深化行政审批制度改革，减少和规范行政审批事项。加强政府信息公开，扩大政务公开范围，保障人民群众的知情权、参与权和监督权。加快电子政务建设，强化目标管理、政务督查工作，完善行政问责制度。加强公务员队伍教育管理，深入开展争做"人民满意的公务员"活动，进一步提高服务效能。

　　（三）坚持依法行政。严格按照法定权限和程序行使权力、履行职责，严格执行市人大及其常委会的决议、决定，依法接受其法律监督和工作监督，主动接受人民政协的民主监督，支持政协参政议政，认真办理人大代表建议和政协提案。加强法治政府建设，坚持依法民主决策，严格文明执法。支持工会、共青团、妇联等人民团体发挥桥梁纽带作用。重视人民群众通过法定渠道对政府机关的监督。支持监察、审计等部门依法独立履行职责。重视发挥新闻舆论和社会公众的监督作用。

　　（四）做到廉洁为民。严格执行党风廉政建设责任制和领导干部廉洁从政若干准则，完善惩治和预防腐败体系，严厉查处贪污腐败、失职渎职等违纪违法行为，坚决纠正损害群众利益的不正之风。牢固树立宗旨意识，始终坚持以人为本，不断加强政府规范化、标准化、精细化管理，切实降低行政成本，把有限的资金和资源更多地用于发展经济和改善民生，努力实现好、维护好、发展好最广大人民的根本利益。

　　各位代表！回顾过去，我们无愧于历史；展望未来，我们深感机遇难得。三门峡已经进入到一个崭新的发展阶段，站在新起点，实现新跨越，是时代赋予我们的光荣使命和神圣职责。让我们在省委、省政府和市委的正确领导下，进一步解放思想、开拓创新，勤勉尽责、不负重托，为全面建设创新开放、富裕文明、平安和谐、生态宜居的三门峡而努力奋斗！
